# Wuqinxi exercise for mind and balance: Enhancing cognition, fall prevention, and quality of life in older adults with mild cognitive impairment

**DOI:** 10.1371/journal.pone.0346490

**Published:** 2026-05-06

**Authors:** Qingpan Wen, Shiying Chen, Huan Zhou, Yangjun Liu, Jianye Li, Marcin Białas, Dominika Wilczyńska

**Affiliations:** 1 Faculty of Physical Education, Gdansk University of Physical Education and Sport, Gdańsk, Poland; 2 Faculty of Social and Humanities, WSB Merito University Gdansk, Gdańsk, Poland; Niigata University of Health and Welfare: Niigata Iryo Fukushi Daigaku, JAPAN

## Abstract

**Background:**

The risk of falls is a global public health issue, with over 38 million disability-adjusted life years lost annually due to falls. However, older adults with mild cognitive impairment (MCI) are more likely to fall and suffer more severe injuries compared to cognitively normal older adults, which also has an impact on their quality of life.

**Methods:**

This study was a randomized, controlled trial with two parallel groups, allocated in a 1:1 ratio using a concealed allocation mechanism and assessor blinding. 53 participants were randomly assigned to the 12-week Wuqinxi exercise group or the 12-week stretching exercise group. Overall cognitive function, risk of falls, and quality of life were assessed at baseline and at post. Independent t-tests and non-parametric tests were used to compare the outcome variables between the two groups.

**Results:**

There were no significant differences in baseline demographic characteristics or assessment indicators between Wuqinxi exercise group and stretching exercise group (P > 0.05), indicating comparability between the groups. After 12 weeks of intervention, the Wuqinxi exercise group showed significant improvements in primary outcome measures, including cognitive function and risk of falls (P < 0.001). In the SF-12 quality of life, Wuqinxi exercise group showed statistically significant improvements in six dimensions (P < 0.05), including general Health (GH), physical Function (PF), role Physical (RP), body Pain (BP), role Emotional (RE), and mental Health (MH). In two dimensions insignificant vitality (VT, P = 0.649) and social function (SF, P = 0.089). The median and interquartile range after intervention were also overall better in the Wuqinxi exercise group. In addition, the Mini-Mental State Examination (MMSE) was significantly negatively correlated with Timed Up and Go TestTimed Up and Go Test (TUG) (r = −0.52, p < 0.01) and significantly positively correlated with Modified Falls Efficacy Scale (MFES) (r = 0.463, p < 0.05). The improvement in quality of life in the BP and SF dimensions was significantly positively correlated with the increase Montreal Cognitive Assessment (MoCA) and MMSE respectively (BP-MoCA: r =  0.406, p < 0.05; SF-MMSE: r = 0.399, p < 0.05).

**Conclusion:**

The Wuqinxi exercise is a feasible and acceptable intervention for improving cognitive function, preventing falls, and enhancing quality of life in older adults with MCI. Our study’s findings emphasize the importance of Wuqinxi exercise in older adults’ health management and confirm the feasibility of a large-scale andomized controlled trial.

## Introduction

Mild cognitive impairment (MCI) is often used to refer to the transition stage between normal cognitive function and clinically possible Alzheimer’s disease (AD) [1]. The prevalence of MCI in adults over 65 years old is 3% − 19% [2]. Over time, some elderly people with MCI have stable or recovered cognitive function, but more than 50% of them will progress to AD within five years [3]. The incidence of MCI varies across different living conditions. Overall, the incidence of MCI among Chinese people aged 60 and above is 15.5% [4]. Among 802 elderly residents in Huangpu District, Shanghai, who were surveyed, the incidence of MCI was 31.8% [5]. Among 1,064 elderly people in rural Shanxi, the incidence of MCI was 17.6% [6]. If MCI is not intervened in time, the decline in cognitive function may affect lower limb function (such as walking speed [7,8] and balance ability [9]).

Muscle weakness and impaired balance are well-studied risk factors for falls. [10]. The lower limb muscle strength and postural stability of the elderly are closely related [11,12]. Weak lower limb strength is regarded as a significant risk factor for falls among older adults [13–15]. However, a weakened sense of balance is one of the important factors contributing to the increased risk of falls among the elderly [16,17]. According to an estimate by the World Health Organization, there are 684,000 fatal fall injuries annually, and more than 38 million disability-adjusted life years are lost each year due to falls, making it the second largest non-intentional cause of death after road traffic injuries [18]. Approximately 15% of people aged 65 and over have fallen at least once in the past 12 months [19]. People with MCI have a higher risk of falling [20,21]. Compared with cognitively normal elderly people, MCI increases the risk of falling by more than two times [20,22], which indicates that people with MCI have a higher risk of falling and face more dangerous situations. Consequently, their quality of life will be seriously affected, placing heavy burdens on individuals and families. Taking effective interventions for MCI with older adults may significantly reduce the incidence of dementia. According to a new study published in the Journal of the American Medical Association, exercise intervention is the most effective method for reducing the risk of falling in older adults [23]. Integrating exercise interventions can improve the literature on cognition and quality of life. Older adults are not suitable for high-intensity or complex exercises, whereas traditional Chinese Qigong is a slow, low-intensity exercise suitable for the elderly [24,25].

We plan to conduct a prospective randomized controlled trial (RCT) to investigate the effects of a Wuqinxi exercise program lasting 12-week on cognitive function, fall of risk, and quality of life in older adults with MCI. This study provides strong theoretical support for the choice of non-pharmacological intervention options for older adults with MCI and lays a foundation for the promotion and application of Wuqinxi exercise.

## Materials and methods

### Trial design

This single-center randomized controlled trial was approved by the Ethics Review Committee of Chengdu Sport University (2024#94) and registered on the Chinese Clinical Trial Registry (ChiCTR, http://www.chictr.org.cn) with the registration number ChiCTR2500100001. The study was conducted in accordance with the Declaration of Helsinki. Before the study began, all subjects and their families were informed of the nature, purpose, potential risks and benefits of the study. Oral explanations are provided in the appropriate dialect, and written informed consent is obtained after full understanding.

### Participants

The study Subjects were a community of older adults with MCI. The recruitment event was held in July 2024 on Dashiqiao Street in Dongpo District, Sichuan Province, China. Through community chats, flyer distribution, and the setup of recruitment booths at events. The recruitment will be conducted both online and offline.

**Fig 1:**
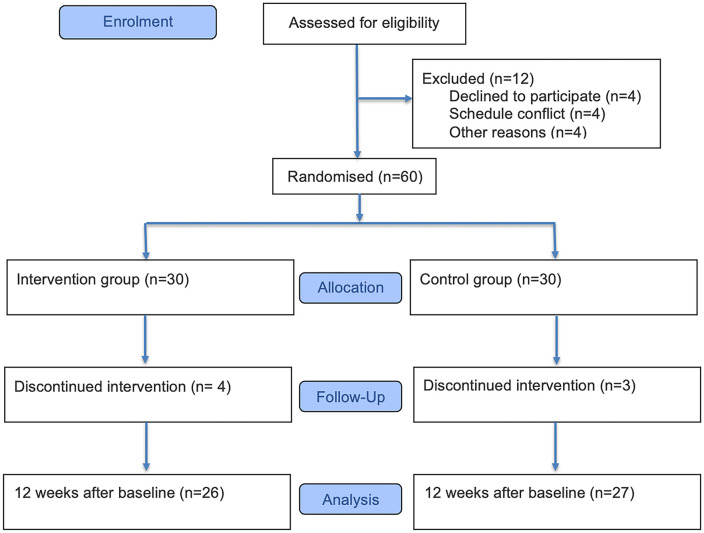
CONSORT flow diagram of participants through the trial.

We analyzed following the principle of the preferred program set (PPT). The 72 residents were surveyed using the questionnaire, which was based on the inclusion and exclusion criteria. After the initial screening, 12 participants withdrew, leaving 60. The reasons for withdrawal were unrelated to the study: 4 refused random allocation, 4 did not specify a reason, and 4 had scheduling conflicts. In the first week of the experiment, 7 participants withdrew, with 4 from the Wuqinxi exercise group (3 due to advanced age and 1 due to a time conflict) and 3 from the stretching exercise group (all due to advanced age). The 53 eligible participants were randomly allocated to the Wuqinxi exercise group (N = 26) or the stretching exercise group (N = 27). The research protocol is shown in Fig 1 and supporting CONSORT checklist.

### Selection criteria

(1) The clinical doctor’s diagnosis or compliance with MCI diagnostic criteria [26];
(2) Chinese older adults age 60 or above, with no significant differences in physical status and basic communication skills;
(3) The Lawton (1969) [27] ADL scale (14–56 points) uses a score of < 26 points [9]; the MoCA (0–30 points) uses a score of ≤ 26 points [28]; the MMSE (0–30 points) uses a score of 18–23 points [29].
(4) No regular exercise (defined as at least 3 times per week for at least 20 minutes each time) and able to perform a moderate amount of physical activity;
(5) No unhealthy habits (no excessive drinking or smoking) and no organic disease;
(6) Informed consent and voluntary participation in screening.

### Exclusion criteria

(1) Patients with poorly controlled hypertension (systolic blood pressure > 160 mmHg or diastolic blood pressure > 100 mmHg);
(2) Those taking anti-dementia drugs;
(3) Those with severe cognitive impairment, mental illness, other neurodegenerative diseases (such as Parkinson’s disease, stroke, frontotemporal dementia, vascular dementia, Lewy body dementia), drug or alcohol addiction, depression, severe cerebral trauma, or severe internal diseases;
(4) Those with severe muscle disorders such as myasthenia gravis, severe fractures, severe arthritis, or those who have had lower-limb injuries within the past 6 months and have difficulty standing or standing for long periods of time, and who cannot participate in the Wushu exercise training;
(5) Severe aphasia or visual and auditory impairments, severe organ failure, a history of coronary artery disease, musculoskeletal diseases, or other exercise contraindications;
(6) Those who participate in medical professional cognitive intervention and regularly practice tai chi, yoga, square dancing (at least 30 minutes per session, at least 3 times a week, for about 1 year), etc.

### Randomization, allocation concealment, and blinding

After completing the baseline assessment, eligible participants were randomly assigned to the WEG group (N = 26) and the SEG group (N = 27). A simple 1∶1 randomization technique was used. The simple random allocation sequence was generated by the randomization program of SPSS (version 26.0, Almonk, New York, NY, USA) and managed by an independent research assistant who was not involved in participant recruitment, evaluation, or intervention. Participants, exercise coaches, and intervention supervisors could not be blinded, but the result evaluators and data statisticians will be blinded to the group allocation. To ensure blinding effectiveness, random allocation was conducted by an independent researcher who was not involved in the study. The allocation results were concealed in sealed, opaque envelopes provided to the researchers before the intervention measures were applied. The intervention duration for both groups was 12 weeks.

### Training protocol

a) **The Wuqinxi Exercise Group**

**Fig 2:**
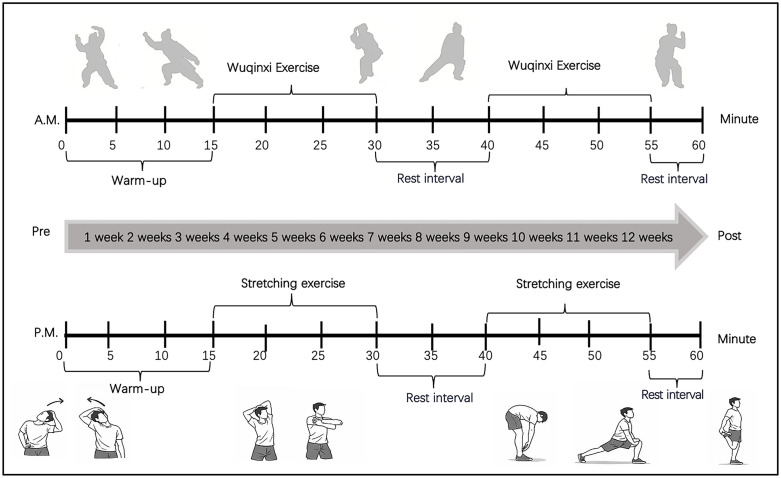
The overall intervention procedu. Notes: **A.**M, *Ante Meridiem.* **P.**M, *Post Meridiem.*

The Wuqinxi Exercise consists of 60-minute sessions, 3 times per week for 12 weeks. Each session includes a 15-minute warm-up, a 40-minute Wuqinxi Exercise, and a 5-minute relaxation phase. The exercise is based on the “ Wuqinxi Exercise Standards” issued by the General Administration of Sport of China in 2020, and consists of (1) tiger raising and seizing, (2) deer colliding and running, (3) bear swaying and rubbing, (4) ape being alert and plunking, and (5) bird stretching and flying. The Wuqinxi Exercise is guided and supervised by professional coaches. A coach from Chengdu Sports University was hired to guide participants in their exercise. A research staff member also attended all training sessions to record each participant’s attendance. If participants encounter any problems during their daily exercise, they should promptly communicate with doctors and coaches. The training protocol is shown in Fig 2.

b) **The stretching Exercise Group**

The stretching exercise consisted of 60-minute sessions, 3 times per week for 12 weeks. Each session included a 15-minute warm-up, 40 minutes of stretching exercise, and a 5-minute relaxation phase. The stretching exercise targeted the upper body (neck, arms, back, shoulders, and chest) and the lower body (quadriceps, hamstrings, calves, and glutes). Before each session, participants were required to perform a warm-up exercise. After the stretching session, participants were required to perform a relaxation exercise. A coach from Chengdu Sports University was hired to guide participants through the exercise. Participants should communicate with doctors and coaches if they encounter any problems during the process and follow the doctor’s advice. The training protocol is shown in Fig 2.

### Outcome measures

a) **Montreal Cognitive Assessment Scale**

The Montreal Cognitive Assessment (MoCA) is a brief (approximately 10-minute) test that assesses an individual’s overall cognitive function and is primarily used to screen elderly individuals who report subjective cognitive decline [28]. The scale includes 11 check items in 7 cognitive domains: visual-spatial and executive function, naming, memory, attention, language, abstraction, delayed recall, and orientation. The total score ranges from 0 to 30, with a maximum of 30 and a normal score of ≥26 points [29]. If the score is < 26, the individual is considered to have MCI. When the years of education are ≥ 12, the total score is increased by 1 point. The Cronbach’s alpha coefficient for the Chinese version of MoCA is 0.818 [30].

b) **Mini-Mental State Examination Scale**

The Mini-Mental State Examination (MMSE) was developed by Folstein et al. in 1975 and is one of the most influential standardized cognitive status examination tools for assessing cognitive impairment [31]. The MMSE assesses 7 aspects: temporal orientation, spatial orientation, immediate memory, attention and calculation, delayed memory, language, and visual-spatial ability. The MMSE score ranges from 0 to 30. The MMSE score is strongly influenced by educational level, so different screening thresholds are used for older adults with varying levels of education [32]. If the total score falls below the threshold, cognitive function may be impaired. MMSE scores of 17–19 (illiterate), 20–22 (primary school), and 24–26 (junior high school or above) are classified as MCI. The test-retest reliability of the instrument was 0.78, and the inter-rater reliability was 0.99 [33].

c) **Five-Times Sit-to-Stand Test**

The Five-Times Sit-to-Stand Test (FTSST) was used to assess the functional mobility of older adults during daily activities [34]. The FTSST may effectively predict the risk of falls among older adults [35]. The test is easy to perform, and the chair is adjusted to each participant’s height and secured against the wall. After the researcher explains the requirements and precautions to the participants, they are asked to cross their hands in front of their chests, keep their feet flat on the ground, and lean back against the chair without touching the backrest. Upon hearing the “start” command, participants are asked to perform the sit-to-stand action as quickly as possible five times, and the time required to complete the five repetitions is recorded. The test can be performed three times, with a 1-minute rest between each. Test-retest and inter-rater reliability were high, with an intraclass correlation coefficient (ICC) ≥ 0.99 [36].

d) **Timed Up and Go Test**

The Timed Up and Go Test (TUG) is a commonly used functional test for balance and gait assessment [37]. Participants were asked to wear their usual shoes and sit on a backless chair with armrests (chair height approximately 45 cm, armrest height approximately 20 cm), with their back against the backrest and their hands on the armrests. A clearly visible thick line was drawn on the ground 3 meters from the chair as a marker. When the researcher gave the command “Start,” the participant stood up, stood still, walked forward 3 meters, passed the marker, turned around, walked back to the chair, turned around, and sat down with their back against the backrest. No physical assistance was provided to the participants during the test [38]. Before the formal test, participants were allowed to practice 1–2 times to ensure a smooth test process. Three tests were conducted, and the average was calculated. TUG has excellent test-retest reliability, with ICC ranging from 0.80 to 0.85 [39,40].

e) **Berg Balance Scale**

The Berg Balance Scale (BBS) is a test used to assess functional balance [41]. The scale comprises 14 items, including transitioning from sitting to standing, independent standing, independent sitting, transitioning from standing to sitting, bed-to-chair transfer, closed-eye standing, and standing with feet together. The higher the score, the stronger the patient’s balance and autonomous postural control. A score below 40 indicates a risk of falling. A score of 41–56 indicates excellent balance function and a low risk of falling, allowing the patient to walk independently. A score of 21–40 indicates some balance ability and a moderate risk of falling, allowing the patient to walk with assistance. A score of 0–20 indicates poor balance function and a high risk of falling, requiring the use of a wheelchair. It achieves an ICC of 0.97 [36,42].

f) **Modified Falls Efficacy Scale**

The Modified Falls Efficacy Scale (MFES) has been widely used in the field of fall risk assessment for older adults, both domestically and internationally [43]. The scale includes 14 items, comprising 9 indoor daily activities (getting in and out of bed, dressing, bathing, reaching into a box or drawer, getting up from a chair, walking around the room, answering the door or the phone, preparing a simple meal, and doing light housework) and 5 outdoor activities (climbing stairs, crossing the street, using public transportation, simple shopping, and light gardening or hanging clothes out to dry). Each item is scored from 0 to 10, with 0 indicating complete inability, 5 indicating average, and 10 indicating good. The final score is the average of all item scores. The MFES demonstrates excellent reliability (ICC = 0.93) and internal consistency (α = 0.95) [44].

g) **12-Item Short-Form Health Survey (SF-12)- Quality of Life Assessment**

The SF-12 has been widely used in clinical research and public health assessments to monitor health outcomes [45]. The SF-12 is a self-administered questionnaire designed to assess the impact of health status on daily activities [46]. The 12-item short form (SF-12) yields 8 subscales: General Health (GH), Physical Function (PF), Role Physical (RP), Body Pain (BP), Role Emotional (RE), Mental Health (MH), Vitality (VT), and Social Function (SF). On the scale, raw scores for each item range from 1 to 6, and participants’ scores are counted and evaluated. In this study, the 8 SF-12 subscales were analyzed.

### Sample size

The sample size was calculated based on the expected improvement in the MoCA score. According to the published literature [47], the mean score differences and standard deviations between the mind-body exercise group and the control group after the intervention were 24.55, 2.25 and 22.35, 1.96 points, respectively, and the effect size was 1.04266. We expected that after a 12-week Wuqinxi exercise intervention, the effect size would be at least 0.2. Using G-Power 3.1.9.6, we calculated a sample size sufficient to detect the target effect size, with a type 1 error of 5% (α = 0.05) and a power of 90% (β = 0.10). Accounting for a 10% dropout rate, a total of 42 participants were needed, with 21 per group.

### Statistical analysis

All statistical analyses were conducted using SPSS (version 26.0, IBM Corp., Armonk, NY, USA). Descriptive statistics summarized demographic characteristics. The t-test compared the means of continuous variables between the two groups, and the chi-square test or Fisher’s exact test assessed the homogeneity of proportions between the two groups. For normally distributed data, a two-tailed independent t-test was used to assess the intervention effect on the main variables. For non-normally distributed data, the Mann-Whitney U test was used. In addition, Pearson correlation analysis (for normally distributed data) or Spearman rank correlation analysis (for non-normally distributed data) was used to explore associations among cognitive function, fall of risk, and quality of life improvement. All hypothesis tests were two-sided, with a significance level of α = 0.05. Data analysis was performed by a statistician on the objective results.

## Results

### Participant characteristics

Table 1 showed the baseline distribution of sociodemographic characteristics for participants in the Wuqinxi exercise group and the stretching control group. A total of 53 patients with MCI were enrolled: the intervention group comprised 16 males (61.5%) and 10 females (38.5%), while the control group comprised 13 males (48.1%) and 14 females (51.9%). Regarding age, 17 participants (65.4%) in the intervention group and 16 (59.3%) in the control group were 65–74 years old. Occupational status showed that 21 participants (80.8%) in the intervention group and 20 (74.1%) in the control group reported manual labor as their primary occupation. Educational attainment was predominantly at the elementary school level: 20 participants (76.9%) in the intervention group and 19 (70.4%) in the control group. Marital status showed that 19 participants (73.1%) in the intervention group and 22 (81.5%) in the control group were married. Regarding retirement allowance, 17 participants (65.4%) in the intervention group and 18 (66.7%) in the control group reported monthly incomes of ¥0-¥1,000. No statistically significant differences were observed between groups for any of these sociodemographic variables (p > 0.05), confirming successful baseline balance.

**Table 1:** Descriptive statistics of demographic characteristics of the Wuqinxi exercise group and stretching exercise group(N = 53).

Variable		WEG (N. = 26)	SEG (N. = 27)	P value
Gender	Men	16（61.5%）	13（48.1%）	0.328
	Female	10（38.5%）	14（51.9%）	
Age	60-64	8(30.8%)	8(29.6%)	0.589
	65-74	17(65.4%)	16(59.3%)	
	75-84	1(3.8%)	3(11.1%)	
Employment status	Intellectual work	5（19.2%）	7（25.9%）	0.560
	Manual work	21（80.8%）	20（74.1%）	
Education	Elementary School	20（76.9%）	19（70.4%）	0.852
	Junior high school	4（15.4%）	5（18.5%）	
	High school or above	2（7.7%）	3（11.1%）	
Marital status	Married	19（73.1%）	22（81.5%）	0.465
	Other	7（26.9%）	5（18.5%）	
Retirement allowance	0-1000	17(65.4%)	18(66.7%)	0.885
	1000-3000	6(23.1%)	5(18.5%)	
	More than 3000	3(11.5%)	4(14.8%)	

WEG: Wuqinxi exercise group; SEG: Stretching exercise group.

Data were presented as the number of participants (percentage).

### MMSE and MoCA: pre and post intervention

Table 2 shows that both Wuqinxi and stretching exercises improve scores on the MoCA and MMSE. Improvements in all indicators in the Wuqinxi exercise group were significantly greater than those in the stretching exercise group (P < 0.001). Specifically, the average improvement values in the intervention group were: MoCA 1.54 ± 0.65, MMSE 2 ± 0.85. In contrast, the control group showed smaller improvements: MoCA 0.52 ± 0.51, MMSE 0.44 ± 0.51. The median and interquartile range after intervention were also overall better in the Wuqinxi exercise group. In conclusion, Wuqinxi exercise is superior to stretching for improving cognitive function in older adults with MCI.

**Table 2:** MMSE and MoCA: Comparison between pre- and post-intervention groups (N = 53).

Variable	Group	Pre-test		Post-test		Post-pre test		t/z	P value
		Mean±SD	Median (IQR)	Mean±SD	Median (IQR)	Mean±SD	Median (IQR)		
MoCA	WEG (N = 26)	19.73 ± 1.56	19（3）	21.27 ± 1.4	21（2）	1.54 ± 0.65	1(1)	−4.94	P < 0.001
	SEG (N = 27)	20.11 ± 2.17	19(3)	20.63 ± 2.06	20(3)	0.52 ± 0.51	1(1)		
MMSE	WEG (N = 26)	22.04 ± 1.34	22(2.25)	24.04 ± 1.11	24(3)	2 ± 0.85	2(2)	−6.65	P < 0.001
	SEG (N = 27)	22.59 ± 1.55	22(3)	23.04 ± 1.43	22(2)	0.44 ± 0.51	0(1)		

WEG: Wuqinxi exercise group; SEG: Stretching exercise group; MMSE: Mini-mental state examination scale; MoCA: Montreal cognitive assessment scale; IQR: Interquartile Range.

### TUG, FTSST, BBS, and MFES: pre and post intervention

Table 3 shows that both Wuqinxi and stretching exercises improve scores on the BBS, TUG, FTSST, and MFES. Improvements across all indicators in the Wuqinxi exercise group were significantly greater than those in the stretching exercise group (P < 0.001). Specifically, the average improvement values for the intervention group were: BBS 4.73 ± 0.67, TUG 0.94 ± 0.12, FTSST 1.92 ± 0.12, and MFES 1.1 ± 0.14. In contrast, the control group showed smaller improvements: BBS 0.48 ± 0.51, TUG 0.37 ± 0.14, FTSST 0.58 ± 0.2, and MFES 0.41 ± 0.15. The median and interquartile range after intervention were also overall better in the Wuqinxi exercise group. In conclusion, Wuqinxi exercise is superior to stretching exercise in improving overall fall-prevention ability among older adults with MCI.

**Table 3:** BBS, TUG, TCS, MFES: Comparison between pre- and post-intervention groups (N = 53).

Variable	Group	Pre-test		Post-test		Post-pre test		t/z	P value
		Mean±SD	Median (IQR)	Mean±SD	Median (IQR)	Mean±SD	Median (IQR)		
BBS	WEG (N = 26)	47.46 ± 0.99	47(1)	52.19 ± 1.33	52(2)	4.73 ± 0.67	5(1)	−6.45	P < 0.001
	SEG (N = 27)	47.59 ± 1.05	48(1)	48.07 ± 1.17	48(2)	0.48 ± 0.51	0(1)		
TUG	WEG (N = 26)	10.21 ± 0.33	10.19(0.36)	9.27 ± 0.32	9.24(0.41)	0.94 ± 0.12	0.95(0.19)	−6.25	P < 0.001
	SEG (N = 27)	10.25 ± 0.25	10.27(0.39)	9.88 ± 0.34	9.96(0.54)	0.37 ± 0.14	0.37(0.24)		
FTSST	WEG (N = 26)	8.83 ± 0.51	8.8（0.92)	6.91 ± 0.54	6.78(1.05)	1.92 ± 0.12	1.92（0.17)	−6.25	P < 0.001
	SEG (N = 27)	8.93 ± 0.51	8.86（0.9)	8.36 ± 0.57	8.27（1.05)	0.58 ± 0.2	0.59（0.24)		
MFES	WEG (N = 26)	6.59 ± 0.79	6.64（1.09)	7.69 ± 0.87	7.72（1.38)	1.1 ± 0.14	1.08（0.23)	−6.26	P < 0.001
	SEG (N = 27)	6.76 ± 0.87	6.71（1.43)	7.17 ± 0.91	7.07（1.64)	0.41 ± 0.15	0.42（0.15)		

WEG: Wuqinxi exercise group; SEG: Stretching exercise group; BBS: Berg Balance Scale; TUG: Timed up and go test; FSTS: Five-times sit-to-stand test; MFES: Modified falls efficacy scale; IQR: Interquartile Range.

### Quality of Life (SF-12): pre and post intervention

Table 4 shows that both Wuqinxi and stretching exercises improve the quality of life score. The Wuqinxi exercise group showed significantly greater improvements across all six dimensions of quality of life (general Health, physical Function, role Physical, body Pain, role Emotional, and mental Health) than the stretching exercise group (P < 0.05). Specifically, the average improvement values in the intervention group were: GH 9.54 ± 5.56, PF 4.31 ± 4.47, RP 4.25 ± 3.89, BP 3.52 ± 4.94, RE 5.81 ± 4.03, MH 4.92 ± 5.46. In contrast, the control group showed smaller improvements: GH 3.19 ± 5.02, PF 1.19 ± 2.06, RP 1.19 ± 2.06, BP 0.76 ± 2.72, RE 2.28 ± 3.56, MH 2.03 ± 3.78. The median and interquartile range after the intervention were also overall better in the Wuqinxi exercise group. There were no significant differences between the two groups in the vitality and social function (P = 0.649 and P = 0.089, respectively). In conclusion, Wuqinxi exercise has a better overall effect than stretching exercise in improving the quality of life of older adults with MCI.

**Table 4:** Quality of Life (SF-12): Comparison between pre- and post-intervention groups (N = 53).

Variable	Group	Pre-test		Post-test		Post-pre test		t/z	P value
		Mean±SD	Median (IQR)	Mean±SD	Median (IQR)	Mean±SD	Median (IQR)		
GH	WEG (N = 26)	49.55 ± 5	51.21(0)	59.09 ± 4.88	61.99(10.78)	9.54 ± 5.56	10.78(0)	−3.79	P < 0.001
	SEG (N = 27)	48.81 ± 5.46	51.21(10.98)	52.01 ± 6.64	51.21(0)	3.19 ± 5.02	0(10.78)		
PF	WEG (N = 26)	49.86 ± 5.6	47.88(8.59)	54.16 ± 4.11	57.18(9.22)	4.31 ± 4.47	2.7(8.59)	−4.43	P < 0.001
	SEG (N = 27)	50.52 ± 2.33	52.57(4.61)	51.72 ± 3.84	52.57(9.22)	1.19 ± 2.06	0(4.61)		
RP	WEG (N = 26)	49.91 ± 2.66	47.96(4.61)	54.16 ± 4.11	57.18(9.22)	4.25 ± 3.89	4.61(9.21)	−3.06	P = 0.002
	SEG (N = 27)	50.52 ± 2.33	52.57(4.61)	51.72 ± 3.84	52.57(9.22)	1.19 ± 2.06	0(4.61)		
BP	WEG (N = 26)	49.21 ± 4.1	47.25(0)	52.74 ± 5.18	50.48(10.19)	3.52 ± 4.94	0(10.19)	−2.42	P = 0.016
	SEG (N = 27)	49.52 ± 5.16	47.25(10.19)	50.27 ± 4.74	47.25(10.19)	0.76 ± 2.72	0(0)		
RE	WEG (N = 26)	44.47 ± 3.85	44.9(1.4)	50.27 ± 3.7	50.49(1.4)	5.81 ± 4.03	5.59(0)	−4.25	P < 0.001
	SEG (N = 27)	45.54 ± 4.2	44.9(5.59)	47.8 ± 3.59	44.9(5.59)	2.28 ± 3.56	0(5.59)		
MH	WEG (N = 26)	51.41 ± 2.83	52.35(0)	56.34 ± 4.54	55.4(6.1)	4.92 ± 5.46	6.1(7.62)	−2.16	P = 0.031
	SEG (N = 27)	50.77 ± 4	52.35(0)	52.8 ± 3.35	52.35(0)	2.03 ± 3.78	0(6.1)		
VT	WEG (N = 26)	60.52 ± 4.55	57.81(10.7)	62.07 ± 5.07	57.81(10.7)	1.55 ± 3.7	0(0)	−0.46	P = 0.649
	SEG (N = 27)	60.79 ± 5.45	57.81(10.07)	61.91 ± 5.04	57.81(10.07)	1.12 ± 3.32	0(0)		
SF	WEG (N = 26)	44.14 ± 4.39	46.47(2.52)	48.8 ± 4.34	46.47(2.52)	4.66 ± 6.53	0(10.1)	−1.70	P = 0.089
	SEG (N = 27)	44.23 ± 4.28	46.47(0)	46.1 ± 4.41	46.47(0)	1.87 ± 4	0(0)		

WEG: Wuqinxi exercise group; SEG: Stretching exercise group; GH: general health; PF: Physical function; RP: Role physical; BP: Body pain; RE: Role-emotional; MH: Mental health; VT: Vitality; SF: Social function; IQR: Interquartile Range.

### The correlation between risk of falls, changes in quality of life, and cognitive function improvement in wuqinxi exercise group

**Fig 3:**
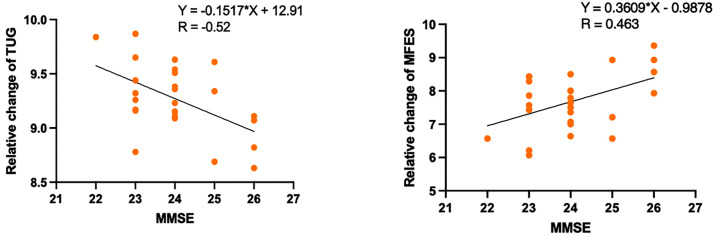
Results showing a significant positive correlation between Risk of Falls and the improvement of Cognitive Function (MMSE) score. Notes: TUG, Timed up and go test. MMSE, Mini-mental state examination scale. MFES, Modified Falls Efficacy Scale.

**Fig 4:**
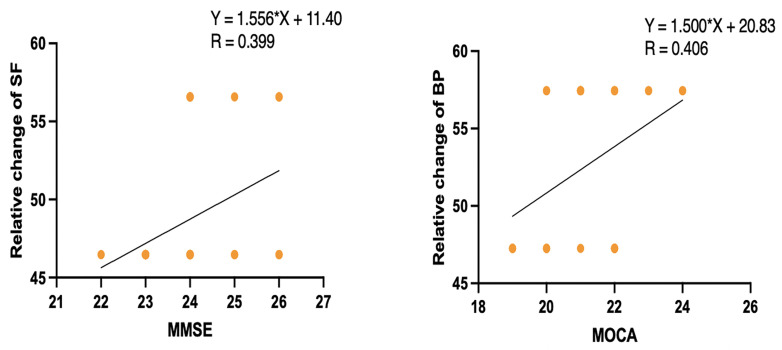
Results showing a significant positive correlation between Quality of Life (SF, BP) and the improvement of Cognitive Function (MMSE and MoCA) score. Notes: BP, Body Pain*.* SF, Social Function. MoCA, Montreal cognitive assessment scale*.* MMSE, Mini-mental state examination scale*.*

Table 5 and Fig 3 show a significant positive correlation between the risk of falls (MFES) and improvement in cognitive function (MMSE score) (r = 0.463, P < 0.05). Table 5 and Fig 3 also show a significant negative correlation between the risk (TUG) and improvement in cognitive function (MMSE score) (r = −0.52, P < 0.01). Table 5 and Fig 4 show a significant positive correlation between quality of life (SF) and improvement in cognitive function (MMSE score) (r = 0.399, P < 0.05). In addition, Table 5 and Fig 4 show a positive correlation between changes in quality of life (BP) and improvement in cognitive function (MoCA score) (r = 0.406, P < 0.05). However, there was no significant correlation between cognitive function and quality of life (GH, PF, RP, RE, MH, VT).

**Table 5:** Correlation between cognitive function, risk of falls, and changes in quality of life.

Variable	MMSE	MoCA
BBS	0.103	0.122
FTSST	−0.35	−0.121
TUG	−0.520**	−0.352
MFES	0.463*	0.207
GH	0.339	0.119
PF	0.196	0.025
RP	0.268	0.114
BP	0.245	0.406*
RE	−0.106	−0.075
MH	0.113	−0.175
VT	0.327	0.172
SF	0.399*	0.172

MMSE: Mini-mental state examination scale; MoCA: Montreal cognitive assessment scale; BBS: Berg Balance Scale; TUG: Timed up and go test; FSTS: Five-times sit-to-stand test; MFES: Modified Falls Efficacy Scale; GH: General Health; PF: Physical Function; RP: Role Physical; BP: Body Pain; RE: Role Emotional; MH: Mental Health; VT: Vitality; SF: Social Function. *Significant at p < 0.05; **significant at p < 0.01.

## Discussion

This study examined the effects of Wuqinxi exercise on cognitive function, risk of falls, and quality of life in older adults with MCI. Wuqinxi exercise, a traditional Chinese Qigong exercise that emphasizes the integration of mind and body, is considered an effective method for promoting both physical and mental health [48]. For middle-aged and older adults, Wuqinxi exercise may promote psychological health by regulating the nervous and hormonal systems, thereby improving overall health [49]. The findings indicate that Wuqinxi exercise may improve cognitive function, risk of falls, and enhance quality of life in older adults. The main finding was that participants with MCI showed significant improvements in cognitive function after 12 weeks of Wuqinxi exercise. In comparison, the improvements in the stretching exercise group were relatively weak. Furthermore, participants in the Wuqinxi exercise group also showed improved risk of falls and quality of life.

Wuqinxi exercise, as a multimodal intervention, integrates physical exercise, cognitive engagement (such as memory and attention required to imitate animal movements), breathing regulation, and mind-body balance. Wuqinxi is a feasible non-pharmaceutical public health strategy that may help slow cognitive decline and reduce the risk of falls among older adults. In clinical practice, incorporating Wuqinxi into the standard care plan for older adults with MCI under professional guidance may help maintain long-term cognitive and physical benefits. In summary, Wuqinxi shows statistically significant and clinically meaningful improvements, supporting its use as an effective non-pharmaceutical approach to delaying cognitive decline, reducing the risk of falls, and enhancing the quality of life of this population.

Throughout the exercise intervention, MCI participants did not experience any adverse reactions, and all participants expressed satisfaction with the program. Our cognitive (MoCA) scores were consistent with those reported by Jing Jin (2020) [50]. These results indicate that, compared with older adults who participated in other interventions, those who practiced qigong experienced less age-related cognitive decline [49,51,52]. We found a strong correlation between the risk of falls (TUG, MFES) and cognitive function (MMSE), suggesting that qigong may be effective in improving cognitive function and reducing the risk of falls.

In this study, the exact mechanism underlying the improvement in cognitive function was unclear. During qigong exercises, the sustained state of relaxation may reduce pro-inflammatory factors such as IL-6 [53], thereby alleviating neuroinflammation through anti-inflammatory effects and providing protection for cognitive function. Qigong exercises may improve cardiopulmonary function and promote neuroplasticity (activating the prefrontal cortex, enhancing memory and attention) [54–56], thereby enhancing cognitive performance. Given the link between physical and mental health, improvements in Mood or reductions in chronic disease symptoms may benefit mental health [57,58]. The dual challenge of physical and mental exercise may increase the burden on physiological and neurophysiological processes, prompting the brain to make positive adaptations [50]. Previous studies have shown that the effects of qigong exercise on older adults are concentrated on overall cognitive function [59,60]. Epidemiological surveys suggest that cognitive dysfunction may lower people’s quality of life and sleep quality [61]. These studies focus solely on the cognitive function of older adults with MCI and do not address other indicators of MCI, such as lower limb strength, balance, and quality of life. Another result of our study found that, in addition to improving measured cognitive function, participants in the Wuqinxi exercise group also showed reduced risk of falls and improved quality of life.

The risk of falls in the Wuqinxi Exercise group improved, and there was a significant positive correlation between the risk of falls (MFES) and cognitive function (MMSE) scores (r = 0.463, P < 0.05). At the same time, this study observed a significant negative correlation between the risk of falls (TUG) and cognitive function (MMSE) scores (r = −0.52, P < 0.01). Additionally, we found that the Wuqinxi Exercise may significantly improve quality of life in this study. The improvement in quality of life was associated with improvements in cognitive function in older adults with MCI. This result is consistent with the findings of other scholars [9]. Changes in cognitive function (MoCA) were significantly positively correlated with quality of life (BP) (r = 0.406, P < 0.05). Changes in cognitive function (MMSE) were also significantly positively correlated with quality of life (SF) (r = 0.399, P < 0.05).

### Limitations of the study

First, we have clearly reported the dropout rate in the article and acknowledged that PPS analysis may overestimate the effect of implementation in a broader population. The present study only inferred the possible mechanisms of the Wuqinxi Exercise on cognitive function, risk of falls, and quality of life through indirect indicators. However, it did not explain the mechanisms of the central nervous system or the biological indicators.

Second, the risk of falls indicators used in the present study were all results of single-task tests. These results reflect interactions among perception, behavior, and cognitive processes. Single-task tests may not fully evaluate or reflect the level of fall-prevention function. Combining multi-task state measurements with single-task state measurements may more effectively measure and reflect the level of fall-prevention function. Moreover, the present study used subjective assessment scales (such as the SF-12) as the outcome indicators. These are personal and limited by human factors and external influences. As an intervention, exercise cannot be ignored by the subjects.

Finally, there were baseline differences in dropout, with 7 participants dropping out. The present study lacked follow-up after the intervention, and it was unable to determine whether the Wuqinxi Exercise would maintain its effect on cognitive function in the long term. There is no further research at present, and the mechanism of impact is unclear. Although this study has revealed many new angles for further research, the current research results still have limitations.

In future research, on the one hand, the effectiveness in the real world should be verified by using the ITT analysis with a larger sample size. On the other hand, the potential functions of this exercise pattern through more detailed central nervous system mechanisms and biological indicators to understand why the Wuqinxi Exercise may cause changes in the cognitive function, risk of falls, and quality of life of older adults with MCI. Because this study is a small sample of exercise intervention research, it has reference value for the launch of new clinical intervention methods.

## Conclusions

In summary, this study shows that 12-week Wuqinxi exercise may improve overall cognitive function in older adults with MCI. Moreover, improvements in overall cognitive function in older adults with MCI may affect the level of risk of falls and quality of life. Wuqinxi exercise is an effective, safe, and beneficial exercise that may improve physical and mental health for different populations. In the future, more attention should be paid to non-pharmacological combinations or to the lowest effective doses of drug interventions that may confer health benefits across different populations.

## Supporting information

S1 Data Baseline Data. (XLSX)

S2 Data Post Data. (XLSX)

S1 File CONSORT checklist. (DOCX)

## References

[1] Winblad B, Palmer K, Kivipelto M, Jelic V, Fratiglioni L, Wahlund L-O, et al. Mild cognitive impairment--beyond controversies, towards a consensus: report of the International Working Group on Mild Cognitive Impairment. J Intern Med. 2004;256(3):240–6. doi: 10.1111/j.1365-2796.2004.01380.x 15324367

[2] Gauthier S, Reisberg B, Zaudig M, Petersen RC, Ritchie K, Broich K, et al. Mild cognitive impairment. Lancet. 2006;367(9518):1262–70. doi: 10.1016/S0140-6736(06)68542-5 16631882

[3] Wang LZY. Early recognition of mild cognitive impairment and research progress of relevant theoretical models. Chinese Journal of Nursing. 2018;53(5). doi: 10.3761/j.issn.0254-1769.2018.05.021

[4] Jia L, Du Y, Chu L, Zhang Z, Li F, Lyu D, et al. Prevalence, risk factors, and management of dementia and mild cognitive impairment in adults aged 60 years or older in China: a cross-sectional study. Lancet Public Health. 2020;5(12):e661–71. doi: 10.1016/S2468-2667(20)30185-7 33271079

[5] Fengqian Q, Weihua C, na GS, Lihua H, Yunfang J, Juan D, et al. A study on influencing factors for mild cognitive impairment in the elderly in Huangpu District, Shanghai. Geriatrics & Health Care. 2024;30(2):403–409,417. doi: 10.3969/j.issn.1008-8296.2024.02.026

[6] Xiao-xia C, Zhia-lan Y, Li-ping C, Hui G, Wei-fang J. Current status and influencing factors of mild cognitive impairment in 1064 elderly people in rural areas of Shanxi. Journal of Nursing. 2023;30(22):7–12. doi: 10.16460/j.issn1008-9969.2023.22.007

[7] Verghese J, Wang C, Lipton RB, Holtzer R, Xue X. Quantitative gait dysfunction and risk of cognitive decline and dementia. J Neurol Neurosurg Psychiatry. 2007;78(9):929–35. doi: 10.1136/jnnp.2006.106914 17237140 PMC1995159

[8] Martin KL, Blizzard L, Wood AG, Srikanth V, Thomson R, Sanders LM, et al. Cognitive function, gait, and gait variability in older people: a population-based study. J Gerontol A Biol Sci Med Sci. 2013;68(6):726–32. doi: 10.1093/gerona/gls224 23112113

[9] Li K, Yu H, Kortas JA, Lin X, Lipowski M. The effect of 12 weeks of Baduanjin exercise on cognitive function, lower limb balance and quality of life of the elderly with mild cognitive impairment: a randomized controlled trial. Gazz Med Ital - Arch Sci Med. 2023;181(11). doi: 10.23736/s0393-3660.22.04802-1

[10] Tinetti ME, Speechley M, Ginter SF. Risk factors for falls among elderly persons living in the community. N Engl J Med. 1988;319(26):1701–7. doi: 10.1056/NEJM198812293192604 3205267

[11] Viitasalo JT, Era P, Leskinen AL, Heikkinen E. Muscular strength profiles and anthropometry in random samples of men aged 31–35, 51–55 and 71–75 years. Ergonomics. 1985;28(11):1563–74. doi: 10.1080/00140138508963288

[12] Lindle RS, Metter EJ, Lynch NA, Fleg JL, Fozard JL, Tobin J, et al. Age and gender comparisons of muscle strength in 654 women and men aged 20-93 yr. J Appl Physiol (1985). 1997;83(5):1581–7. doi: 10.1152/jappl.1997.83.5.1581 9375323

[13] de Rekeneire N, Visser M, Peila R, Nevitt MC, Cauley JA, Tylavsky FA, et al. Is a fall just a fall: correlates of falling in healthy older persons. The Health, Aging and Body Composition Study. J Am Geriatr Soc. 2003;51(6):841–6. doi: 10.1046/j.1365-2389.2003.51267.x 12757573

[14] Wolfson L, Judge J, Whipple R, King M. Strength is a major factor in balance, gait, and the occurrence of falls. J Gerontol A Biol Sci Med Sci. 1995;50 Spec No:64–7. doi: 10.1093/gerona/50a.special_issue.64 7493221

[15] Lord SR, McLean D, Stathers G. Physiological factors associated with injurious falls in older people living in the community. Gerontology. 1992;38(6):338–46. doi: 10.1159/000213351 1473733

[16] Wolfson LI, Whipple R, Amerman P, Kleinberg A. Stressing the postural response. A quantitative method for testing balance. J Am Geriatr Soc. 1986;34(12):845–50. doi: 10.1111/j.1532-5415.1986.tb07256.x 3782696

[17] Wolfson L, Whipple R, Derby CA, Amerman P, Murphy T, Tobin JN, et al. A dynamic posturography study of balance in healthy elderly. Neurology. 1992;42(11):2069–75. doi: 10.1212/wnl.42.11.2069 1436514

[18] Falls OW. Available. https://www.who.int/zh/news-room/fact-sheets/detail/falls. 2024. Accessed 2024 November 28.

[19] Zhang L, Ding Z, Qiu L, Li A. Falls and risk factors of falls for urban and rural community-dwelling older adults in China. BMC Geriatr. 2019;19(1):379. doi: 10.1186/s12877-019-1391-9 31888516 PMC6937656

[20] Ansai JH, Andrade LP de, Masse FAA, Gonçalves J, Takahashi AC de M, Vale FAC, et al. Risk Factors for Falls in Older Adults With Mild Cognitive Impairment and Mild Alzheimer Disease. J Geriatr Phys Ther. 2019;42(3):E116–21. doi: 10.1519/JPT.0000000000000135 28786910

[21] Lach HW, Harrison BE, Phongphanngam S. Falls and Fall Prevention in Older Adults With Early-Stage Dementia: An Integrative Review. Res Gerontol Nurs. 2017;10(3):139–48. doi: 10.3928/19404921-20160908-01 27665756

[22] Tyrovolas S, Koyanagi A, Lara E, Santini ZI, Haro JM. Mild cognitive impairment is associated with falls among older adults: Findings from the Irish Longitudinal Study on Ageing (TILDA). Exp Gerontol. 2016;75:42–7. doi: 10.1016/j.exger.2015.12.008 26707711

[23] Liu-Ambrose T, Davis JC, Best JR, Dian L, Madden K, Cook W, et al. Effect of a Home-Based Exercise Program on Subsequent Falls Among Community-Dwelling High-Risk Older Adults After a Fall: A Randomized Clinical Trial. JAMA. 2019;321(21):2092–100. doi: 10.1001/jama.2019.5795 31162569 PMC6549299

[24] Guo Y, Shi H, Yu D, Qiu P. Health benefits of traditional Chinese sports and physical activity for older adults: A systematic review of evidence. J Sport Health Sci. 2016;5(3):270–80. doi: 10.1016/j.jshs.2016.07.002 30356513 PMC6188612

[25] Jahnke R, Larkey L, Rogers C, Etnier J, Lin F. A comprehensive review of health benefits of qigong and tai chi. Am J Health Promot. 2010;24(6):e1–25. doi: 10.4278/ajhp.081013-LIT-248 20594090 PMC3085832

[26] Petersen RC, Smith GE, Waring SC, Ivnik RJ, Tangalos EG, Kokmen E. Mild cognitive impairment: clinical characterization and outcome. Arch Neurol. 1999;56(3):303–8. doi: 10.1001/archneur.56.3.303 10190820

[27] Lawton MP, Brody EM. Assessment of older people: self-maintaining and instrumental activities of daily living. Gerontologist. 1969;9(3):179–86. doi: 10.1093/geront/9.3_part_1.179 5349366

[28] Nasreddine ZS, Phillips NA, Bédirian V, Charbonneau S, Whitehead V, Collin I, et al. The Montreal Cognitive Assessment, MoCA: a brief screening tool for mild cognitive impairment. J Am Geriatr Soc. 2005;53(4):695–9. doi: 10.1111/j.1532-5415.2005.53221.x 15817019

[29] Folstein MF, Folstein SE, McHugh PR. “Mini-mental state”. A practical method for grading the cognitive state of patients for the clinician. J Psychiatr Res. 1975;12(3):189–98. doi: 10.1016/0022-3956(75)90026-6 1202204

[30] Zhang L, Liu X. A study on reliability and validity of MoCA scale of Chinese version. Chinese Nursing Research. 2007;21(31):2906–7.

[31] Gallegos M, Morgan ML, Cervigni M, Martino P, Murray J, Calandra M, et al. 45 Years of the mini-mental state examination (MMSE): A perspective from ibero-america. Dement Neuropsychol. 2022;16(4):384–7. doi: 10.1590/1980-5764-DN-2021-0097 36530763 PMC9745978

[32] Freitas S, Simões MR, Alves L, Santana I. The Relevance of Sociodemographic and Health Variables on MMSE Normative Data. Appl Neuropsychol Adult. 2015;22(4):311–9. doi: 10.1080/23279095.2014.926455 25531579

[33] Chiu HF, Lee HC, Wing YK, Kwong PK, Leung CM, Chung DW. Reliability, validity and structure of the Chinese Geriatric Depression Scale in a Hong Kong context: a preliminary report. Singapore Med J. 1994;35(5):477–80. 7701365

[34] Goldberg A, Chavis M, Watkins J, Wilson T. The five-times-sit-to-stand test: validity, reliability and detectable change in older females. Aging Clin Exp Res. 2012;24(4):339–44. doi: 10.1007/BF03325265 23238309

[35] Weng C, Liu L. The five times sit to stand test: a useful assessment tool for predicting falls in elderly. Chinese Journal of Rehabilitation Medicine. 2012;027(010):908–12. doi: 10.3969/j.issn.1001-1242.2012.10.004

[36] Meseguer-Henarejos A-B, Rubio-Aparicio M, López-Pina J-A, Carles-Hernández R, Gómez-Conesa A. Characteristics that affect score reliability in the Berg Balance Scale: a meta-analytic reliability generalization study. Eur J Phys Rehabil Med. 2019;55(5):570–84. doi: 10.23736/S1973-9087.19.05363-2 30955319

[37] Qin RYJZBLM. The timed up and go test for fall risk prediction in older adults: the latest advances. Chinese Journal of Geriatrics. 2022;41(6):743–7. doi: 10.3760/cma.j.issn.0254-9026.2022.06.026

[38] Chan PP, Si Tou JI, Tse MM, Ng SS. Reliability and Validity of the Timed Up and Go Test With a Motor Task in People With Chronic Stroke. Arch Phys Med Rehabil. 2017;98(11):2213–20. doi: 10.1016/j.apmr.2017.03.008 28392324

[39] Huang S-L, Hsieh C-L, Wu R-M, Tai C-H, Lin C-H, Lu W-S. Minimal detectable change of the timed “up & go” test and the dynamic gait index in people with Parkinson disease. Phys Ther. 2011;91(1):114–21. doi: 10.2522/ptj.20090126 20947672

[40] Steffen T, Seney M. Test-retest reliability and minimal detectable change on balance and ambulation tests, the 36-item short-form health survey, and the unified Parkinson disease rating scale in people with parkinsonism. Phys Ther. 2008;88(6):733–46. doi: 10.2522/ptj.20070214 18356292

[41] Miranda N, Tiu TK. Berg Balance Testing. Treasure Island (FL): StatPearls Publishing. Copyright © 2024, StatPearls Publishing LLC.; 2024. 34662032

[42] Maeda N, Urabe Y, Murakami M, Itotani K, Kato J. Discriminant analysis for predictor of falls in stroke patients by using the Berg Balance Scale. Singapore Med J. 2015;56(5):280–3. doi: 10.11622/smedj.2015033 25678051 PMC4447930

[43] Song J, Yang P, Liu G. The self-rated fall risk questionnaire and modified falls efficacy scale in assessing the fall risk in community-dwelling older Chinese adults: a comparative study. Chinese General Practice. 2022;25(25):3097–3100,3106. doi: 10.12114/j.issn.1007-9572.2022.0225

[44] Hill KD, Schwarz JA, Kalogeropoulos AJ, Gibson SJ. Fear of falling revisited. Arch Phys Med Rehabil. 1996;77(10):1025–9. doi: 10.1016/s0003-9993(96)90063-5 8857881

[45] Gandek B, Ware JE, Aaronson NK, Apolone G, Bjorner JB, Brazier JE. Cross-validation of item selection and scoring for the SF-12 Health Survey in nine countries: results from the IQOLA Project. J Clin Epidemiol. 1998;51(11):1171–8. doi: 10.1016/s0895-4356(98)00109-7 9817135

[46] Ware J Jr, Kosinski M, Keller SD. A 12-Item Short-Form Health Survey: construction of scales and preliminary tests of reliability and validity. Med Care. 1996;34(3):220–33. doi: 10.1097/00005650-199603000-00003 8628042

[47] Tao J, Liu J, Chen X, Xia R, Li M, Huang M, et al. Mind-body exercise improves cognitive function and modulates the function and structure of the hippocampus and anterior cingulate cortex in patients with mild cognitive impairment. Neuroimage Clin. 2019;23:101834. doi: 10.1016/j.nicl.2019.101834 31128522 PMC6535682

[48] Guo Y, Xu M, Wei Z, Hu Q, Chen Y, Yan J, et al. Beneficial Effects of Qigong Wuqinxi in the Improvement of Health Condition, Prevention, and Treatment of Chronic Diseases: Evidence from a Systematic Review. Evid Based Complement Alternat Med. 2018;2018:3235950. doi: 10.1155/2018/3235950 30473716 PMC6220394

[49] Cai L. Review of health effect of Wuqinxi exercise in China. Wushu Studies. 2016;1(12):93–6. doi: 10.13293/j.cnki.wskx.006256

[50] Jin J, Wu Y, Li S, Jin S, Wang L, Zhang J, et al. Effect of 1 Year of Qigong Exercise on Cognitive Function Among Older Chinese Adults at Risk of Cognitive Decline: A Cluster Randomized Controlled Trial. Front Psychol. 2020;11:546834. doi: 10.3389/fpsyg.2020.546834 33192794 PMC7662077

[51] Guo Y, Shi H, Yu D, Qiu P. Health benefits of traditional Chinese sports and physical activity for older adults: A systematic review of evidence. J Sport Health Sci. 2016;5(3):270–80. doi: 10.1016/j.jshs.2016.07.002 30356513 PMC6188612

[52] Chan JSY, Deng K, Wu J, Yan JH. Effects of Meditation and Mind-Body Exercises on Older Adults’ Cognitive Performance: A Meta-analysis. Gerontologist. 2019;59(6):e782–90. doi: 10.1093/geront/gnz022 30796782

[53] Qiu W, Pan H, Wen X, Zhao Z, Jing C, Feng Y. Research on the anti-aging effects of Baduanjin Qigong fitness exercises. Journal of New Chineses in Medicine. 2014;46(7):3. doi: 10.13457/j.cnki.jncm.2014.07.039

[54] Wayne PM, Walsh JN, Taylor-Piliae RE, Wells RE, Papp KV, Donovan NJ, et al. Effect of tai chi on cognitive performance in older adults: systematic review and meta-analysis. J Am Geriatr Soc. 2014;62(1):25–39. doi: 10.1111/jgs.12611 24383523 PMC4055508

[55] Kim THM, Pascual-Leone J, Johnson J, Tamim H. The mental-attention Tai Chi effect with older adults. BMC Psychol. 2016;4(1):29. doi: 10.1186/s40359-016-0137-0 27245444 PMC4886430

[56] Tsang WWN, Chan KK, Cheng CN, Hu FSF, Mak CTK, Wong JWC. Tai Chi practice on prefrontal oxygenation levels in older adults: A pilot study. Complement Ther Med. 2019;42:132–6. doi: 10.1016/j.ctim.2018.11.005 30670231

[57] Abbott R, Lavretsky H. Tai Chi and Qigong for the treatment and prevention of mental disorders. Psychiatr Clin North Am. 2013;36(1):109–19. doi: 10.1016/j.psc.2013.01.011 23538081 PMC3917559

[58] Xia R, Wan M, Lin H, Ye Y, Chen S, Zheng G. Effects of mind-body exercise Baduanjin on cognition in community-dwelling older people with mild cognitive impairment: A randomized controlled trial. Neuropsychol Rehabil. 2023;33(8):1368–83. doi: 10.1080/09602011.2022.2099909 35838817

[59] Ladawan S, Klarod K, Philippe M, Menz V, Versen I, Gatterer H, et al. Effect of Qigong exercise on cognitive function, blood pressure and cardiorespiratory fitness in healthy middle-aged subjects. Complement Ther Med. 2017;33:39–45. doi: 10.1016/j.ctim.2017.05.005 28735824

[60] Tsang HWH, Lee JLC, Au DWH, Wong KKW, Lai KW. Developing and testing the effectiveness of a novel health qigong for frail elders in Hong Kong: a preliminary study. Evid Based Complement Alternat Med. 2013;2013:827392. doi: 10.1155/2013/827392 24109493 PMC3784263

[61] Reynolds CF 3rd, Frank E, Perel JM, Imber SD, Cornes C, Miller MD, et al. Nortriptyline and interpersonal psychotherapy as maintenance therapies for recurrent major depression: a randomized controlled trial in patients older than 59 years. JAMA. 1999;281(1):39–45. doi: 10.1001/jama.281.1.39 9892449

